# Supplementary material for: A defined syphilis vaccine candidate inhibits dissemination of Treponema pallidum subspecies pallidum
Source: Nat Commun. 2017 Feb 1;8:14273. doi: 10.1038/ncomms14273 (PMC5296639; doi:10.1038/ncomms14273)
Supplement: Supplementary Information — Supplementary Figures, Supplementary Tables and Supplementary Reference [file ncomms14273-s1.pdf]

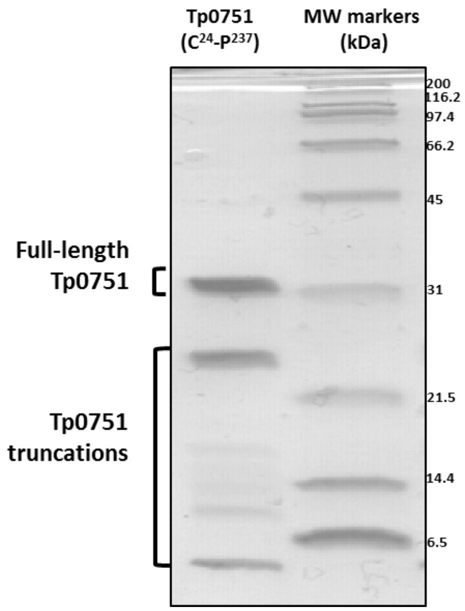

Supplementary Figure 1. Recombinant Tp0751 preparation used for rabbit immunization. SDS-PAGE of the recombinant Tp0751 (C^{24}-P^{237}) preparation used as an antigen for rabbit immunizations. As previously confirmed with mass spectrometry and Edman sequencing[1], full-length Tp0751 corresponds to the 31 kDa band, while remaining bands represent truncated forms of the protein.

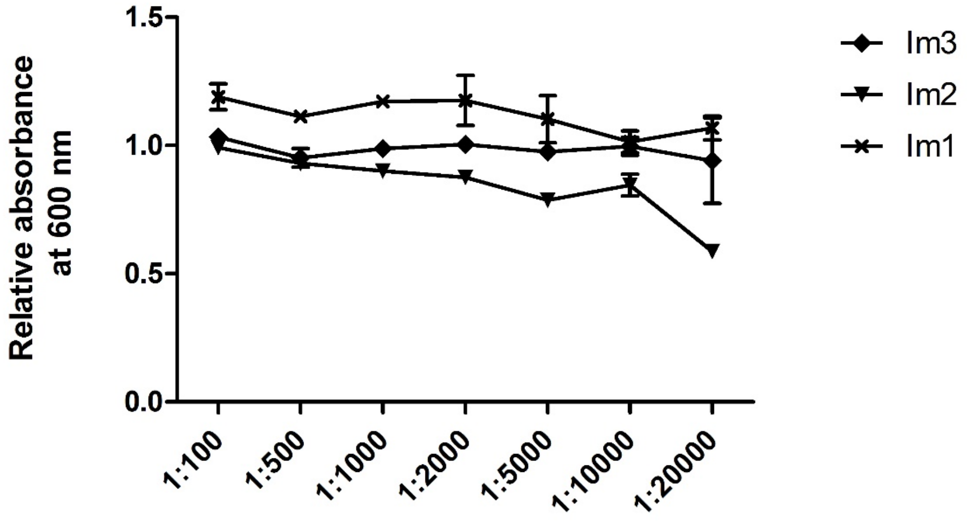

Supplementary Figure 2. Rabbit serum titers against recombinant Tp0751 antigen. Blood samples from each immunized animal were collected seven days after the third immunization/second boost and prior to intradermal challenge with *T. pallidum.* Serum titers were established for the immunized animals Im1, Im2, and Im3 via enzyme-linked immunosorbent assays (ELISAs) with the recombinant Tp0751 antigen. Reactive serum was detected using goat anti-rabbit IgG HRP antibody and TMB (3,3',5,5'-Tetramethylbenzidine) peroxidase substrate and absorbance reading at 600 nm. Titers are displayed relative to serum reactivity from rabbit Im26, another Tp0751-immunized animal from an independent experiment used to generate specific antiserum.

Supplementary Table 1. Serological analysis of animals challenged with *T. pallidum.*

	Animal	Test	Pre-Bleed	Terminal Bleed Day 14
Control	Ct1	VDRL	NR	R 1:4, WR 1:8
	Ct2	VDRL	NR	R 1:4, WR 1:8
Immunized	Im1	VDRL	NR	R 1:2, WR 1:4
	Im2	VDRL	NR	R 1:8, WR 1:16
	Im3	VDRL	NR	R1:2, WR 1:4

NR: non-reactive

R: reactive

WR: weakly reactive

Supplementary Table 2. **Serological analysis of naïve recipient animals following popliteal lymph node transfer.**

	Donor Animal	Recipient Animal	Test	Pre-Bleed	Day 17	Day 31	Terminal Bleed Day 39	Terminal Bleed Day 42	Day 51	Day 73	Day 106	Terminal Bleed Day 134*	Day 147	Day 175	Day 185
Control	Ct1	R-Ct1	VDRL	NR	WR 1:2	R 1:4, WR 1:8		R 1:8, WR 1:16							
			FTA-ABS	0	0	2+		4+							
	Ct2	R-Ct2	VDRL	NR	WR 1:2	R 1:2, WR 1:4	R 1:16								
			FTA-ABS	0	0	3+	4+								
Immunized	Im1	R-Im1	VDRL	NR	NR	NR			NR	NR	NR	NR	NR	NR	NR
			FTA-ABS	0	0	0			0	0	0	0	0	0	0
	Im2	R-Im2	VDRL	NR	NR	NR			NR	NR	NR	NR	NR	NR	NR
			FTA-ABS	0	0	0			0	0	0	0	0	0	0
	Im3	R-Im3	VDRL	NR	NR	NR			WR 1:2	R 1:1, WR 1:2	WR 1:1-1:2	R 1:1, WR 1:2			
			FTA-ABS	0	0	1+			2+	3+	4+	4+			

NR: non-reactive

R: reactive

WR: weakly reactive

FTA-ABS scale: 1+, low reactivity; 4+, high reactivity

* R-Im3 was euthanized on day 134

Supplementary Reference:

1. Houston, S., *et al*. Bifunctional role of the *Treponema pallidum* extracellular matrix binding adhesin Tp0751. *Infect. Immun.* **79**, 1386-1398 (2011)
